# Correction: Nrf2 overexpression increases the resistance of acute myeloid leukemia to cytarabine by inhibiting replication factor C4

**DOI:** 10.1038/s41417-022-00519-5

**Published:** 2022-08-29

**Authors:** Tianzhen Hu, Chengyun Pan, Tianzhuo Zhang, Ming Ni, Weili Wang, Siyu Zhang, Ying Chen, Jishi Wang, Qin Fang

**Affiliations:** 1 College of Pharmacy, Guizhou Medical University, Guiyang, Guizhou China; 2 Department of Haematology, Affiliated Hospital of Guizhou Medical University, Guizhou Province Institute of Hematology, Guiyang, Guizhou China; 3 School of Basic Medical Sciences, Guizhou Medical University, Guiyang, Guizhou China; 4 pharmacy department, Affiliated Hospital of Guizhou Medical University, Guiyang, Guizhou China

**Keywords:** Leukaemia, Gene expression

Correction to: *Cancer Gene Therapy* 10.1038/s41417-022-00501-1, published online 15 July 2022

In Table 2 of this article, the data in the Table 2 headed Nrf2 overexpression increases cytarabine resistance in acute myeloid leukemia through inhibition of replication factor C4 were mistakenly listed under the table 2.

**Table 2:** Sample characteristics at before relapse (newly diagnosed) and relapse in the same AML patient (*n* = 15).

		Before relapse	Relapse	*P* Value
WBC (×10^9^/L)	Median	13.74	15.44	0.736
	Range	2.11–223.39	0.66–185.89	
HB (×10^9^/L)	Median	79.00	94.00	0.580
	Range	44.00–129.00	23.00–129.00	
PLT (×10^9^/L)	Median	51.00	24.00	0.623
	Range	4.00–346.00	6.00–402.00	
RBC (×10^9^/L)	Median	2.25	2.63	0.415
	Range	1.27–4.06	1.27–3.85	
BM Blast (%)	Median	42.00	71.75	0.073
	Range	21.29–93.57	22.06–92.14	
Gene mutation	Mutation	4 (26.7%)	6 (40%)	0.456
	No mutation	11 (73.4%)	9 (60%)	

*BM* bone marrow, *HB* hemoglobin, *PLT* platelet and *WBC* white blood cell, *RBC* red blood cell.

We have carefully corrected this error, and the corrected Table 2 is as shown above.

The original article has been corrected.

